# An Investigation of the Cutting Strategy for the Machining of Polar Microstructures Used in Ultra-Precision Machining Optical Precision Measurement

**DOI:** 10.3390/mi12070755

**Published:** 2021-06-27

**Authors:** Chen-Yang Zhao, Chi-Fai Cheung, Wen-Peng Fu

**Affiliations:** 1 School of Mechanical Engineering and Automation, Harbin Institute of Technology, Shenzhen 518055, China; zhaochenyang@hit.edu.cn (C.-Y.Z.); 20s153227@stu.hit.edu.cn (W.-P.F.); 2 Department of Industrial and System Engineering, The Hong Kong Polytechnic University, Hong Kong 999077, China

**Keywords:** polar microstructures, optimization, machining parameters, ultra-precision machining, cutting strategy

## Abstract

In this paper, an investigation of cutting strategy is presented for the optimization of machining parameters in the ultra-precision machining of polar microstructures, which are used for optical precision measurement. The critical machining parameters affecting the surface generation and surface quality in the machining of polar microstructures are studied. Hence, the critical ranges of machining parameters have been determined through a series of cutting simulations, as well as cutting experiments. First of all, the influence of field of view (FOV) is investigated. After that, theoretical modeling of polar microstructures is built to generate the simulated surface topography of polar microstructures. A feature point detection algorithm is built for image processing of polar microstructures. Hence, an experimental investigation of the influence of cutting tool geometry, depth of cut, and groove spacing of polar microstructures was conducted. There are transition points from which the patterns of surface generation of polar microstructures vary with the machining parameters. The optimization of machining parameters and determination of the optimized cutting strategy are undertaken in the ultra-precision machining of polar microstructures.

## 1. Introduction

In precision measurement, many basic applications with optical sensing technologies are applied, such as the laser interferometer principle, piezoelectric actuator principle, and micro-encoding principle [1,2,3,4]. One of their common characteristics is that they have exacting requirements on the environment, such as vacuum and equipment components, so it is necessary to control the correlative uncertainty sources [5]. For example, errors of air wavelengths and refractive index are the essential uncertainty sources [6] and would lead to Abbe and accumulation errors for multiple degree-of-freedom (DOF) tasks, due to such principles only enabling the measurement of a single DOF [7]. To avoid these limitations, vision-based techniques are used for precision measurement [8,9,10,11]. Currently, the vision-based technique is an appealing method for precision measurement because of its technological advantages, including the visualization result, multiple DOF measurement, easy operation and installation, etc. A novel method that attempts to integrate computer vision and ultra-precision machining technologies [11,12,13] has been presented, which is feasible and promising for precision measurement due to its simplicity, space-saving, low-cost, and high-robust features, etc. The template matching algorithm has been employed for image processing and determined the absolute position of the selected image in the global map [11]. Moreover, a unique surface topography named polar microstructure has been developed for the abovementioned measurement method [12,13]. Polar microstructures aim to serve as a unique global map used for the subsequent matching measurement.

**Figure 1:**
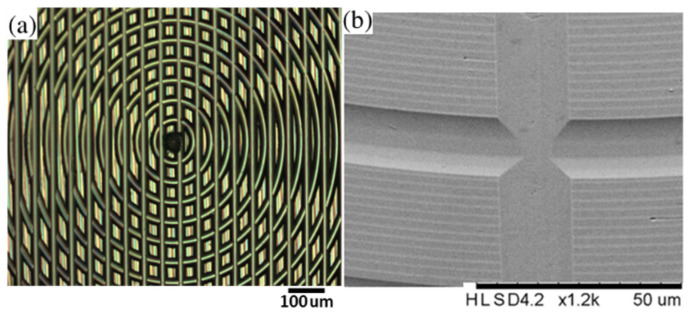
Polar microstructure observed with (**a**) Olympus BX 53M light microscope and (**b**) HITACHI TM3000 Scanning Electron Microscope.

Inspired by the polar coordinate system, a polar microstructure is presented [11], as shown in Figure 1. To generate the polar microstructure surface topography with straight lines and concentric circle trails, a process chain introduced in the literature [14] is designed and fabricated by a combination of single-point diamond turning (SPDT) and single-point diamond grooving (SPDG) processes. It is worth noting that the polar microstructure with form accuracy within the micrometer range still possesses a high discrimination rate. Taking into account the specific characteristics on the surface of the polar microstructure, both the geometric pattern and the arrangement of pixel intensity values are unique. This is due to the unique gray-scale intensity distribution of the polar microstructure that ensures the high reliability and robustness for the optical precision measurement with this polar microstructure.

However, the determination of machining parameters is necessary to fabricate polar microstructures. There are many factors to consider in order to meet the functional performance requirements, which include the parameters for microscopic observation, etc. Currently, there are only a few examples of research concerning the optical resolution of micro-structured surface influenced by cutting strategy [15,16,17,18]. In this paper, an investigation into the influence of machining parameters of polar microstructures to satisfy the extraction of the feature point matching is presented. First, the chosen field of view was investigated. After that, the modeling of polar microstructures is described, and the algorithm for detecting the feature points is explained. In the results and discussion section, three important parameters for the cutting strategy for polar microstructures are identified, which are tool geometry, depth of cut, and groove spacing. The optimized parameters were obtained according to the simulation and experimental results.

## 2. Feature Point Distribution Analysis in the Field of View (FOV)

### 2.1. Determination of the FOV

**Figure 2:**
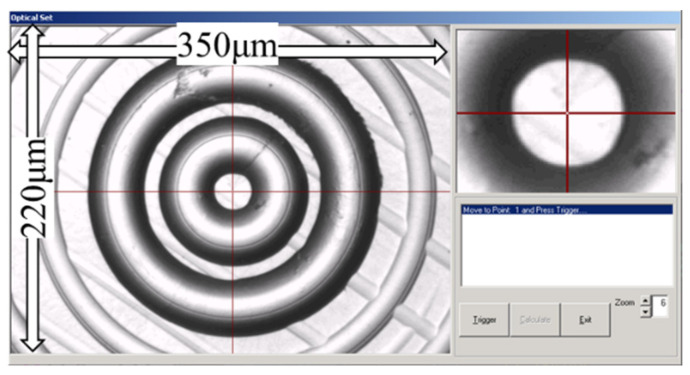
This is a microscope FOV.

The choice of machining parameters takes into account various aspects, such as the field of view (FOV) of the microscope. There are many definitions of FOV, such as the vertical angle or horizontal angle of perspective [19,20,21]. In this paper, FOV refers to the actual size of the area observed by the microscope or camera. The determination of machining parameters of polar microstructure ensures the functional reliability under extreme conditions. Since the FOV of different microscopes or cameras are different, Figure 2 shows a microscope with its FOV matched with the machine tool. This microscope is used to capture the special pattern of polar microstructures for optical position measurement based on the integration of computer vision and ultra-precision machining technologies [11,12,13]. As shown in Figure 2, it is found that its FOV is a rectangular area 350 μm long and 220 μm wide. One design principle is that there are a number of uniform, sufficient, and accurate feature points, which can be detected by feature point detection algorithms.

### 2.2. The Vital Parameters Influencing Feature Point Distribution in the FOV

**Figure 3:**
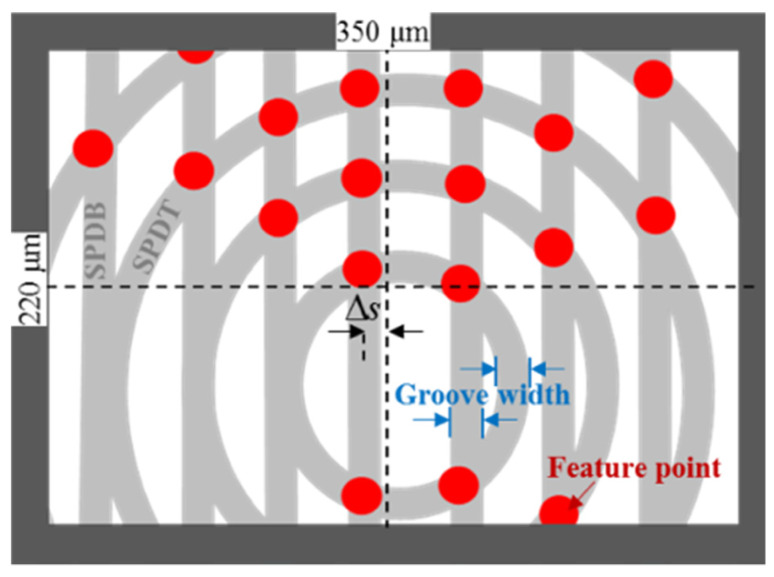
Geometrical relationship between feature point distribution in a microscope’s field of view.

Figure 3 shows the relationship between machining parameters and the FOV. The highlighted intersection points refer to the possible detected designed feature points. Here, it needs to be emphasized that their actual localizations may not be completely consistent with the designed locations for detected feature points (DFP). This is due to the fact that the actual intensity gradient of pixels is not necessarily consistent with the design points due to the machining errors. As a result, the pixels in this area also have differences. However, this does not influence the subsequent matching accuracy since the captured images are from one polar microstructure surface. On the other hand, the measurement algorithms [11] ensure the robustness of the measurement method in response to the pixel intensity changes.

According to the analysis of the designed feature point distribution in the FOV, a polar microstructure was fabricated by SPDT and SPDG, as shown in Figure 3, where the intersection points of the two above machining processes constitute the designed feature points. The distribution of feature points is mainly determined by two aspects, i.e., one is the groove spacing, including both SPDT with SPDG, while the other is the groove width. Furthermore, the generation of the groove width is influenced by the machining parameters, including tool nose radius and depth of cut. Hence, three groups of machining parameters are determined, which are investigated to study their influence on the feature point distribution.

**Figure 4:**
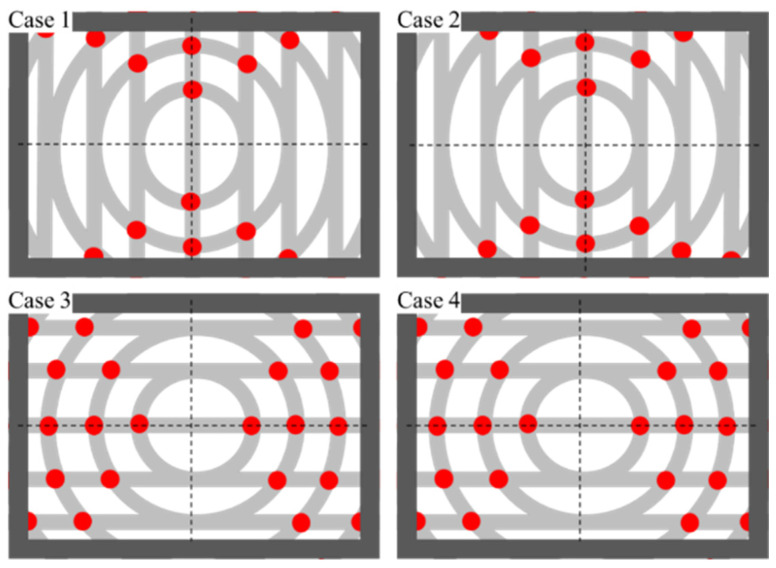
There are four angle detection results without offset (Δs=0) Case1: 0°; Case2: 180°; Case3: 90°; Case4: 270°.

**Figure 5:**
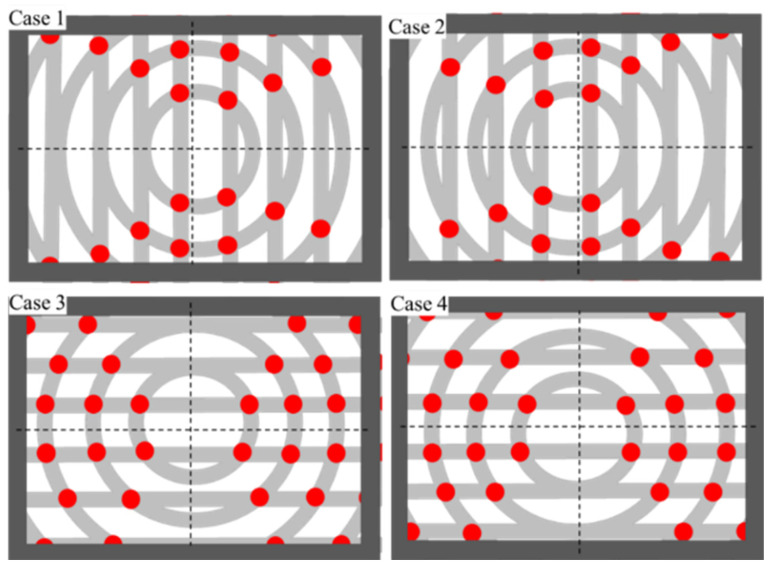
There are four angle detection results with offset (Δs≠0) Case1: 0°; Case2: 180°; Case3: 90°; Case4: 270°.

Another necessary parameter not mentioned before is the offset of straight grooves, which is denoted by the symbol Δs. Its existence ensures the distinction of a 360-degree displacement angle on a polar microstructure surface. For example, Figure 4 shows the polar microstructure without Δs, and the measurable angle range is only 180 degrees. Theoretically, the measurement algorithms cannot distinguish the central symmetrical pattern when it rotates 180 degrees with exactly the same distribution of feature points. However, when there is an offset, the measurable angle range is improved, as shown in Figure 5, which realizes a 360-degree detection range.

## 3. Modeling of Surface Generation for Polar Microstructures

**Figure 6:**
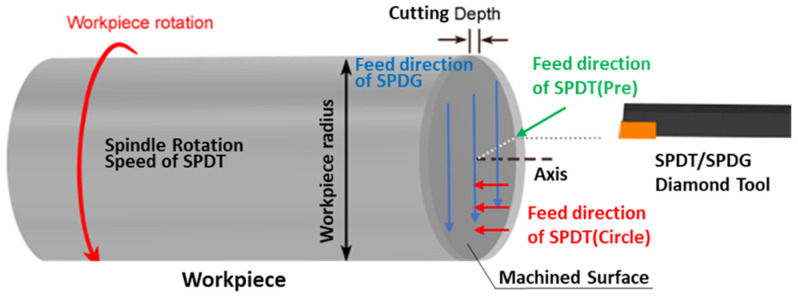
Processing principle of SPDT and SPDG. In the preliminary process of SPDT(Pre), the workpiece turns at a high cutting speed, and the diamond tool feeds in the radial direction until the machined surface becomes smooth. For the concentric circle groove processing in SPDT(Circle), the workpiece keeps spinning, and the diamond tool remains stationary after every certain position of feed direction of SPDT(Circle). For the parallel straight groove processing in SPDG, the workpiece remains stationary and the diamond tool feeds in a series of parallel and equally spaced radial directions.

According to the process chain combining SPDG and SPDT, a polar microstructure is a workpiece with a specific surface microstructure. Figure 6 shows the processing principle of SPDT and SPDG. The workpiece is mounted and rotates on the spindle, and the diamond tool mounted on the machine slides on the machine tool.

**Figure 7:**
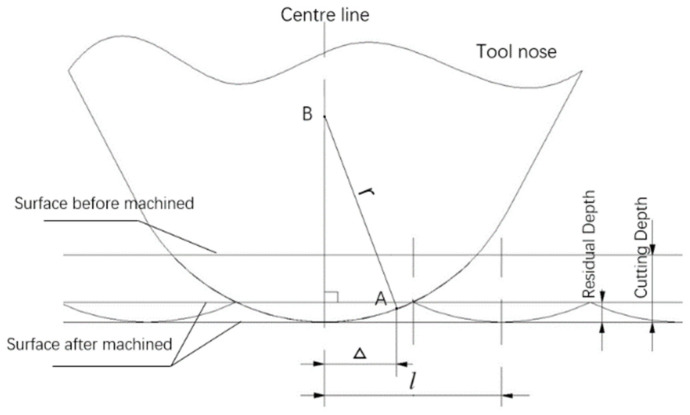
The surface topography geometrical model in SPDT(Pre). l is the length of feed per turn of workpiece; r is the arc tip radius; point $A\left(x_{A},y_{A},z_{A}\right)$ is an arbitrary position on the end surface of the workpiece; $B\left(x_{B},y_{B},z_{B}\right)$ is the center of the tool nose arc; Δ is the distance between the center line of the tool nose and point A.

SPDT(Pre) is an end processing operation that generates the initial workpiece surface, so the initial surface topography model (STM) of the workpiece needs to be firstly established. In this processing operation, the spindle rotational speed for the SPDT is 2000 r/min and the feed rate is 2 mm/min. The geometrical relationship of the initial STM between the tool nose (arc tip) and the initial ST is shown in Figure 7.

**Figure 8:**
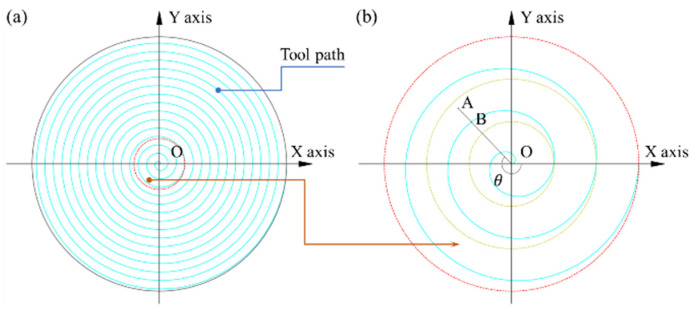
(**a**) Diamond tool path of SPDT(Pre) relative to the surface of the workpiece; (**b**) five-x local magnification view.

Theoretically, the formation of the initial surface topography is related to the tool and cutting parameters, including the arc tip radius, spindle rotational speed, and feed rate. As shown in Figure 7, l can be represented by Equation (1):

(1) $$l=\frac{f}{\omega }$$

where f is the tool feed rate, ω is the spindle rotational speed of the workpiece, and Δ is deduced as Equation (2):

(2) $$\Delta =\left|\sqrt{x_{A}{}^{2}+y_{A}{}^{2}}-k\cdot l\right|$$

where k is the period number from point O to point A. The position relationship between point O and point A is more clearly referred to in Figure 8b. $x_{A}$ and $y_{A}$ are the projecting position coordinates of point A on the diamond tool path plane.

According to the movement of the turning face in SPDT(Pre), the cutting tool path formed is a spiral of Archimedes or uniform speed spiral. The cutting tool path of SPDT(Pre) relative to the surface of the workpiece is shown as Figure 8a. As shown in Figure 8a, point O is the center point of the uniform speed spiral; the five-x magnification views of the red circle in Figure 8a is shown in Figure 8b, which depicts the position relationship between the projecting position of point A and point B; the projecting position of point A is a point on the line OB.

Based on the geometrical relationship, Equations (3)–(5) can be derived as follows:

(3) $$\theta =\arctan\left(\frac{y_{B}}{x_{B}}\right)$$

(4) $$t_{B}=\frac{\theta }{2\pi \left(\frac{\omega }{60}\right)}=\frac{30\theta }{\pi \omega }$$

(5) $$r_{OB}=\frac{f\Delta t_{B}}{60}=k\Delta l$$

where $t_{B}$ is the feeding time of the tool nose between point B and point O and $r_{OB}$ is the polar radius of B. $x_{B}$ and $y_{B}$ are the projecting position coordinates of point B on the diamond tool path plane. θ is the counterclockwise angle from line OX to line OB.

According to the Pythagorean theorem, it is easy to obtain the height $z_{pre}$ of point A, which is derived by Equation (6):

(6) $$z_{pre}=\left(r-d_{1}\right)-\sqrt{r^{2}-\Delta^{2}}$$

where $d_{1}$ is the cutting depth.

The initial STM establishing the workpiece in SPDT(Pre) is accomplished, and the derivation is similar to the STM established in SPDT(Circle) and SPDG(Line). The difference between the model established in SPDT and SPDG is the cutting tool path of the tool nose relative to the workpiece. The tool path of the SPDT(Circle) model is a group of concentric circles. For the SPDG model, the tool path is a series of parallel grooves. To distinguish the parameters, $z_{pre}$, $z_{con}$, and $z_{str}$ represent the workpiece surface height in the SPDT(Pre) model, the SPDT(Circle) model, and the SPDG model, respectively.

Combining the process chain model, the surface topography of the polar microstructure is generated as a consequence of the processing models of SPDT and SPDG. For an arbitrary point on the surface of the workpiece, the surface height $z_{chain}$ results from the minimum height between $z_{pre}$, $z_{con}$, and $z_{str}$, which can be defined as Equation (7):

(7) $$z_{chain}=\arg\underset{\left(x,y\right)\in W}{\min}\left\{z_{pre},z_{con},z_{str}\right\}$$

where W is the workpiece machined area. Hence, the process chain model has been established.

**Figure 9:**
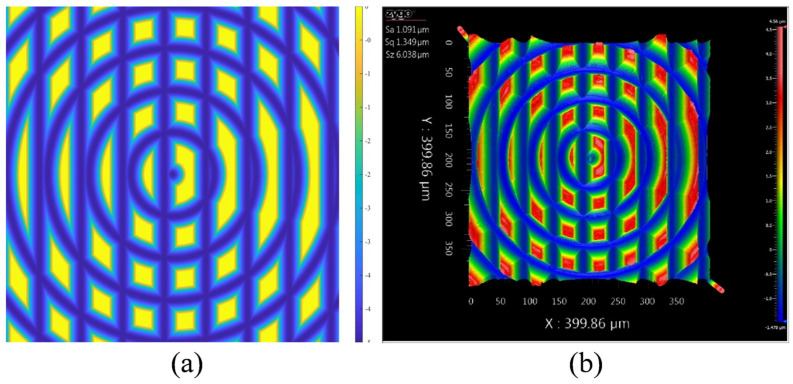
Comparison between modeling and experimental results: (**a**) Simulated surface texture, (**b**) measured surface texture. Machining conditions: Material: Nickel-Copper; Tool radius: 43 μm; Groove spacing: 50 μm; Depth of cut: 5 μm.

According to the models of SPDT and SPDG, simulation experiments under different machining parameters were conducted. Figure 9 shows one of the comparison groups between simulated and experimental results; a high degree of similarity between the two surface texture images is found. The result demonstrates that the proposed model is capable of representing the actual machining conditions of the polar microstructure surface. In other words, the simulated surface topography is able to be used in the further image processing as a substitute of the measured surface images. Furthermore, additional attention should be paid to the simulation model, which is able to reduce the cost and improve the efficiency.

## 4. Feature Point Detection

The modeling of polar microstructures aims to provide their surface topography, which can be shown in the form of images for the feature point detection, which is very significant for the further computer vision-based measurements. The measurement is realized by image matching, and the matching is accomplished by a series of corresponding feature points distributed in different images. As a result, it is necessary to develop the algorithm for feature point detection. There have been many studies providing feature point detection methods, such as canny edge [22], Difference of Gaussians (DoG) [23], and the principal curvature-based region (PCBR) [24]. In this paper, a method named fast and robust feature-based positioning (FRFP) [13] is presented for feature point detection.

Feature point extraction aims to construct a Hessian Matrix (HM) to generate points of interest for feature extraction, named ‘Feature point.’ Constructing the HM aims to find image stable edge points and blob points and provides a basis for the next step of feature extraction. The HM of an image expressed as g(x,y) is given in Equation (8):

(8) $$H\left(g\left(x,y\right)\right)=\left(\begin{array}{cc} \frac{\partial^{2}g}{\partial x^{2}} & \frac{\partial^{2}g}{\partial x\partial y}\\ \frac{\partial^{2}g}{\partial x\partial y} & \frac{\partial^{2}g}{\partial y^{2}} \end{array}\right)$$

The filtered HM by Gaussian filtering is expressed in Equation (9):

(9) $$H\left(g,\sigma \right)=\left(\begin{array}{cc} L_{xx}\left(g,\sigma \right) & L_{xy}\left(g,\sigma \right)\\ L_{xy}\left(g,\sigma \right) & L_{yy}\left(g,\sigma \right) \end{array}\right)$$

**Figure 10:**
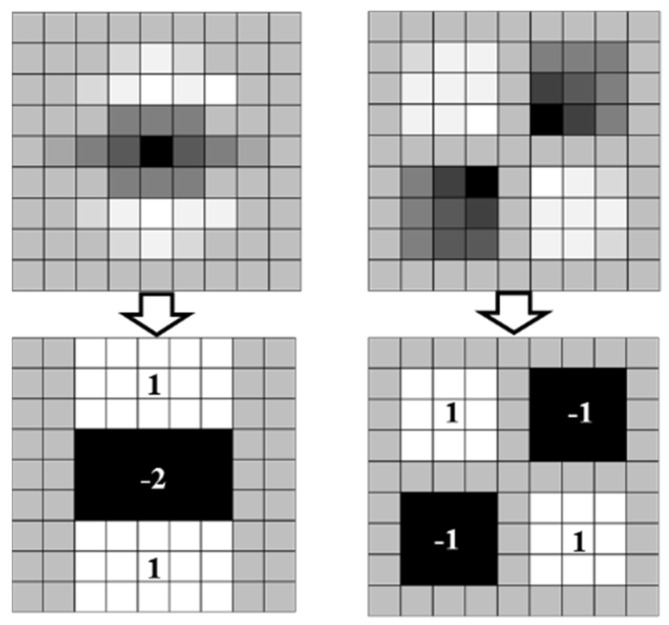
Gaussian filter to box filter.

To increase the speed of the algorithm, this comes up with one box filter (BF) to replace the Gaussian filter (GF). A schematic diagram of a GF and a BF is shown in Figure 10. $D_{xx}$, $D_{yy}$, and $D_{xy}$ are used as the approximation for $L_{xx}$, $L_{yy}$, and $L_{xy}$. The upper two figures, as shown in Figure 10, are the values of the second derivative of the 9 × 9 GB template in the vertical direction on the image, which are $L_{yy}$ and $L_{xy}$, and the lower two images are approximated by using a BF, which are $D_{yy}$ and $D_{xy}$. As shown in Figure 10, the pixel values of the white, black, and gray parts are 1, −2, and 0.

**Figure 11:**
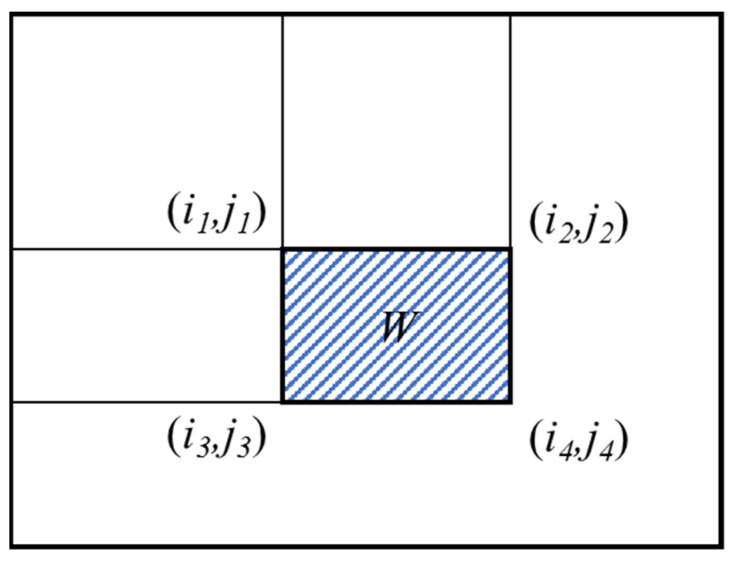
Concept of integral images.

Since the integral image method is used for image convolution, BF increases the computational speed of the algorithm. The integral image method is a fast algorithm that only needs to traverse an image to get the sum of all the pixels in the image, which improves the efficiency of image eigenvalue calculation. The concept of the integral image is shown in Figure 11. For any point (i,j) in an integral image, its value is the sum of the gray values of the rectangular region from the upper-left point of the original image to the point (i,j), which can be expressed as Equation (10):

(10) $$ii\left(i,j\right)=\underset{i^{\prime }\leq i,\;j^{\prime }\leq i}{\sum }p\left(i^{\prime },j^{\prime }\right)$$

The filtering calculated by BF of an image is equal to calculating the pixel sums among the regions of the image, that is, the strength of the integral graph, and can be simply realized by inquiring an integral graph. Since the integral of a region is determined for a given image, the only work that needs to be done is to calculate the values of the four vertices of this region in the integral image, which can be obtained by two-step addition and two-step subtraction. The mathematical formula is expressed in Equation (11):

(11) $$\sum_{W}=ii\left(i_{4},j_{4}\right)-ii\left(i_{2},j_{2}\right)-ii\left(i_{3},j_{3}\right)+ii\left(i_{1},j_{1}\right)$$

When a point is brighter or dimmer than other points surrounding it, the discriminant of the HM yields a local extremum, which determines the position of such a key point. The discriminant of the HM can be derived by Equation (12):

(12) $$\begin{array}{l} \mathrm{Det}\left(H\right)=L_{xx}L_{yy}-L_{xy}L_{xy}\\ =D_{xx}\frac{L_{xx}}{D_{xx}}D_{yy}\frac{L_{yy}}{D_{yy}}-D_{xy}\frac{L_{xy}}{D_{xy}}D_{xy}\frac{L_{xy}}{D_{xy}}\\ =D_{xx}D_{yy}\left(\frac{L_{xx}}{D_{xx}}\frac{L_{yy}}{D_{yy}}\right)-D_{xy}D_{xy}\left(\frac{L_{xy}}{D_{xy}}\frac{L_{xy}}{D_{xy}}\right)\\ =\left(D_{xx}D_{yy}-D_{xy}D_{xy}\left(\frac{L_{xy}}{D_{xy}}\frac{L_{xy}}{D_{xy}}\right)\left(\frac{D_{xx}}{L_{xx}}\frac{D_{yy}}{L_{yy}}\right)\right)\left(\frac{L_{xx}}{D_{xx}}\frac{L_{yy}}{D_{yy}}\right)\\ =\left(D_{xx}D_{yy}-{\left(\omega D_{xy}\right)}^{2}\right)C \end{array}$$

In Equation (12), constant C has no effect on the comparison of key points. In this way, when Gaussian second-order differential filtering is used with σ=1.2 and the template size is 9 × 9 as the smallest scale space value to filter the image, ω in Equation (12) can be expressed in Equation (13):

(13) $$\omega =\frac{{\left|L_{xy}\left(1.2\right)\right|}_{F}{\left|D_{xy}\left(9\right)\right|}_{F}}{{\left|L_{xx}\left(1.2\right)\right|}_{F}{\left|D_{xy}\left(9\right)\right|}_{F}}=0.912$$

Equation (12) is then simplified to Equation (14):

(14) $$\mathrm{Det}\left(H\right)=\left(D_{xx}D_{yy}-{\left(0.192D_{xy}\right)}^{2}\right)$$

where a weighting factor ω=0.912 is used to remedy the error caused by BF approximation. Additionally, a response value is standardized based on the filter size to ensure that the Frobenius norm of the filter of any size is uniform. At a certain image point, its blob response value is represented by the approximate Hessian matrix determinant. A response image of all detected points on a certain scale is formed after all pixel points are traversed. Moreover, taking diverse template sizes obtains a multi-scale blob response map, which is applied to feature point localization.

**Figure 12:**
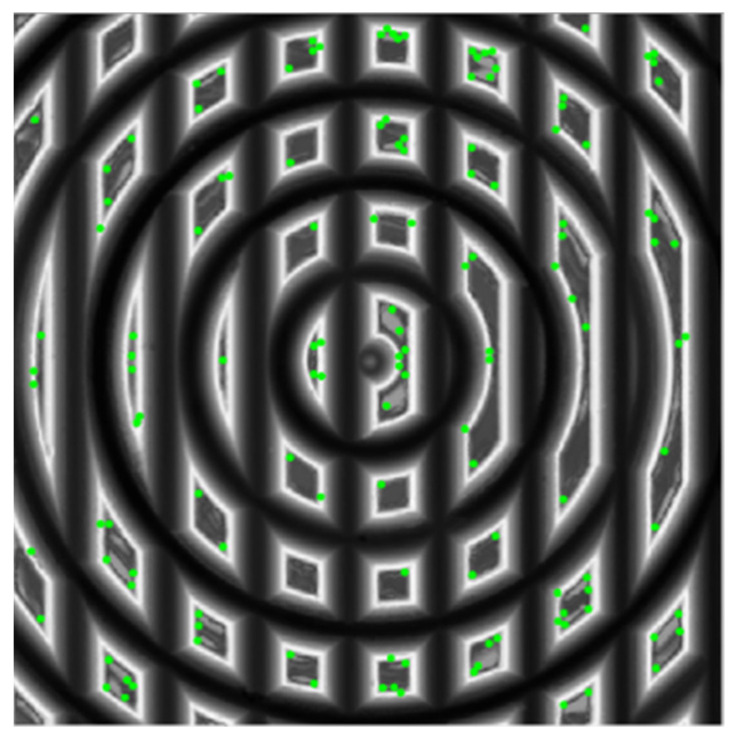
Feature point detection results marked with bright points.

Figure 12 shows a sample of detection results by using FRFP algorithms. It is shown that the main DFP are usually distributed around the designed intersection points, as shown in Figure 3. This demonstrates that the detection algorithm is suitable for accurate detection of the feature points of polar microstructures. However, in order to reduce the localization errors of feature points, the algorithm increases the threshold of detection to ensure that the DFP are accurate and stable enough. On the basis of the above principles, the greater the number of feature points to be detected, the greater the number of subsequent matching points, and the more accurate the final measurement result.

The following section presents an investigation of three machining factors, which are tool nose radius, depth of cut, and groove spacing. For each factor, different machining values of the parameters were simulated to output the surface image of polar microstructures. Feature point detection was then conducted, and the number of detected points was found as the most important criterion for performance evaluation of polar microstructures.

## 5. Results and Discussion

### 5.1. Effect of Tool Geometry

**Figure 13:**
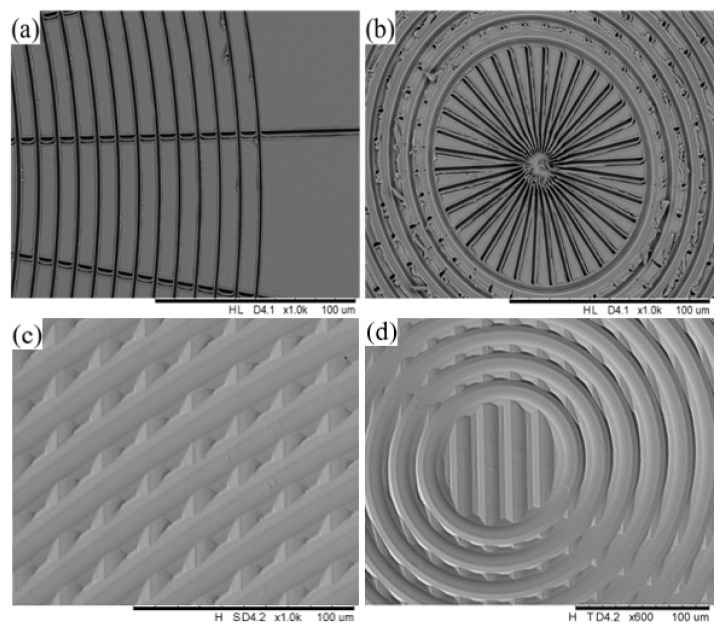
Comparing workpiece machined surfaces by using the sharp tip type and the arc tip type of the diamond tool. Tool wear evolution of the sharp tip type of diamond tool: (**a**) initial machining quality of the workpiece surface by the sharp tip; (**b**) workpiece surface machining quality after tool wear of the sharp tip. Machining quality of the workpiece surface by the arc tip; (**c**) workpiece surface machining quality in the adjacent area; and (**d**) workpiece surface machining quality in the central area.

The reason for firstly studying the influence of tool geometry lies in the actual condition. There are two types of diamond tools; one is the sharp tip and the other is the arc tip. The sharp tip is able to machine a fairly narrow groove width, but the tool wear is the biggest constraint for utilization. Figure 13 shows a case of machining grooves with 10-μm spacing with a sharp tip and a contrasting case with an arc tip, and the workpiece was measured with a scanning electron microscope (SEM). Figure 13a is the initial machining stage, and the machining quality was found to be satisfactory, but the sharp tip was worn out at the end stage, as shown in Figure 13b. Comparatively, the arc tip of the diamond cutting tool was not easily worn out and more suitable for prolonging the tool life for the SPDT and SPDG machining processes, as shown in Figure 13c,d. Hence, the cutting tool type was chosen to be the arc tip.

**Figure 14:**
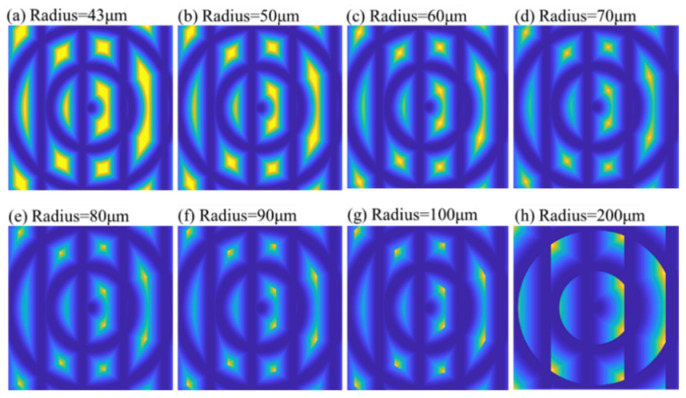
Comparison of simulated surface topography of a polar microstructure with different tool nose radii. Other important machining parameters: groove spacing: 50 μm, depth of cut: 5 μm.

Considering the extreme conditions, polar microstructures should have distinctive patterns and enough accurate feature points in the FOV area of the 200-μm square. As a result, the simulations should be conducted in a 200-μm square area. According to the previous modeling, tool nose radius is one of the parameters for the generation of the polar microstructure surface. In this section, a series of simulations conducted with the different tool nose radius (TNR), ranging from 43 to 200 μm, is presented, and the smallest TNR was set to 43 μm, limited to the experimental conditions. The simulation results are shown in Figure 14.

**Figure 15:**
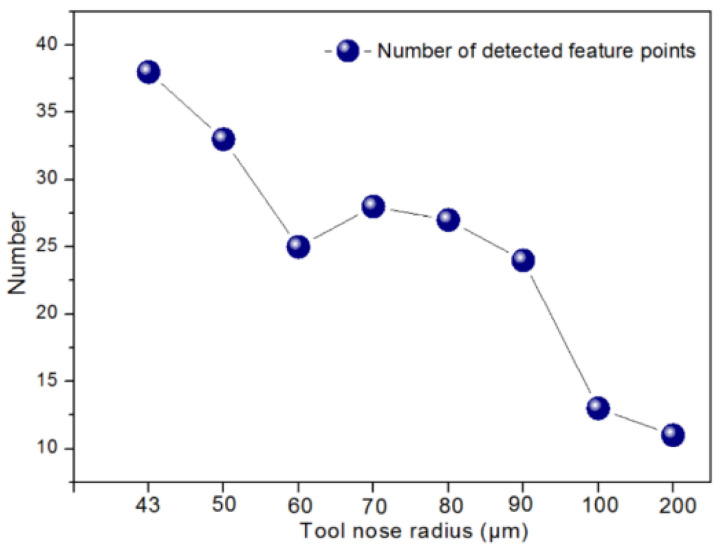
Trend of number of detected feature points (DFP) with different tool nose radii.

With the TNR increasing, the amount of material removal grew, and the groove width became broader. Meanwhile, the intensity change rate of the image weakened, which may reduce the number of DFP. It is worth noting that the number of DFP reduced when the TNR increased, as shown in Figure 15. The number of reduced DFP adversely affected the process of subsequent matching. Hence, it is concluded that smaller TNR is helpful to improve the feature point detection of polar microstructure surface. Considering the current experimental conditions, the smallest TNR (43 μm) can be chosen in future experiments.

### 5.2. Effect of Depth of Cut

**Figure 16:**
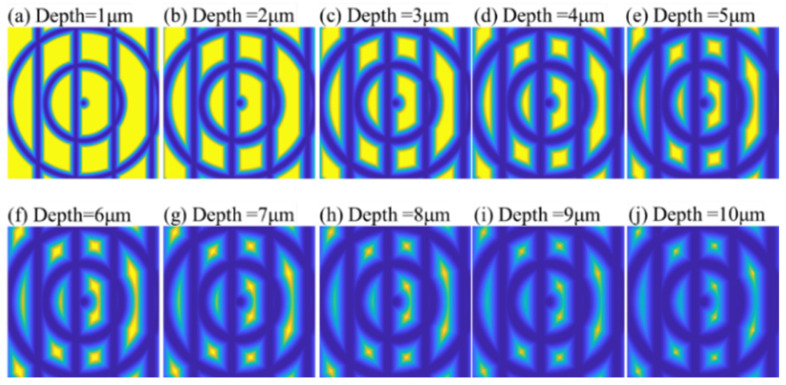
Comparison of the simulated surface topography of a polar microstructure with different depths of cut. Other important machining parameters: groove spacing: 50 μm, tool nose radius: 50 μm.

**Figure 17:**
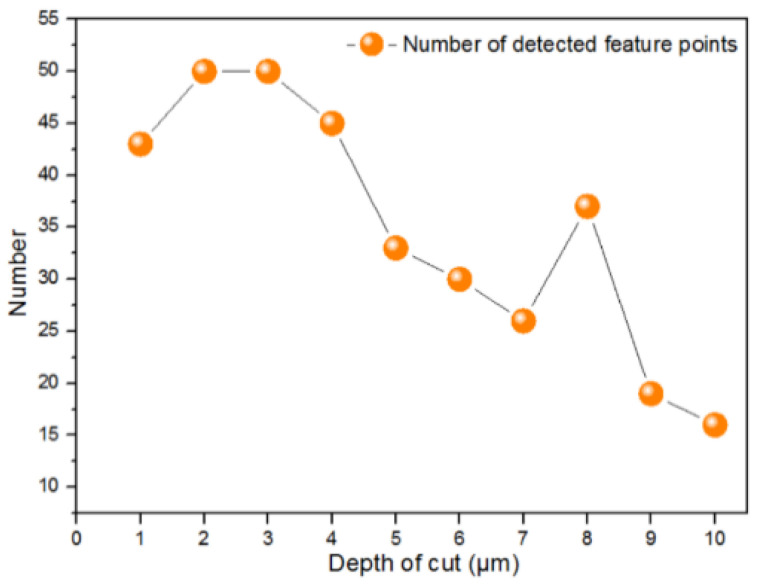
Trend of number of detected feature points (DFP) with different depths of cut.

Besides the TNR, the depth of cut (DOC) also influenced the groove width. Figure 16 shows the simulated results when the DOC was varied. A feature point detection process was also conducted, as shown in Figure 17. Similar to the results for TNR, a smaller DOC was helpful in improving the feature point detection of the polar microstructure surface. However, it is worth noting that there is an increasing number of detected points when the DOC increased from 1 to 2 μm. After comparing the distribution of DFP among these conditions, it was found that the DFP were mainly concentrated in the surrounding area of the highlighted intersection points, as shown in Figure 3. When the DOC was too small, the area of the highlighted intersection points was smaller, and hence the total possible DFP reduced in number.

Moreover, it can be observed in Figure 17 that there was an increase in the number of DFP when the DOC was 8 μm. One of the reasons for this is an algorithm error and uncertainty; the simple central moment is only an algorithm to test the feature point detection ability of polar microstructures; advanced algorithms should be investigated and applied in precision measurement. The other important reason for this is that not all of the DFP could be matched well when the noises of the images were large, such as the scale or angle change, etc., and lead to the number of detected and matched points reducing further; this is also why the number of designed feature points should not be too small. From the view of machining ability, the DOC is usually set manually, and it is difficult to control it accurately when the DOC is too small. To prevent some areas from not perhaps being machined due to the small given DOC, the depth of cut was determined to be 5 μm.

### 5.3. Groove Spacing

In addition to groove width, groove spacing (GS) is an important parameter affecting the performance of polar microstructures. The determination of GS should take into account both functional requirements and actual machining ability. For functional requirements, polar microstructures should have distinctive patterns and enough accurate feature points in the FOV area.

**Figure 18:**
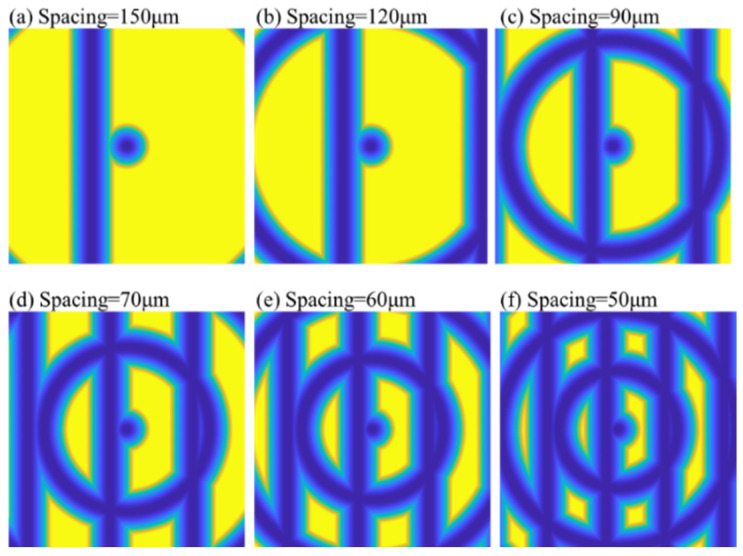
Comparison of simulated surface topography of a polar microstructure with different groove spacing. Other important machining parameters: depth of cut: 5 μm, tool nose radius: 50 μm.

Firstly, a series of simulations was carried out whose GS ranged from 150 μm to 50 μm, with six groups in total. The simulated results are shown in Figure 18. In order to ensure enough feature points against various noises, the number of designed feature points should be large enough. From Figure 18a–e, the number of designed feature points was too small, making it hard to ensure the stability of matching accuracy. Moreover, it cannot be neglected that the simulated area is focused on the central area of polar microstructures, but the designed points for the other areas should also be sufficient and distinctive. Shown by the simulation results analysis, as shown in Figure 18, the GS should not be larger than 50 μm.

**Figure 19:**
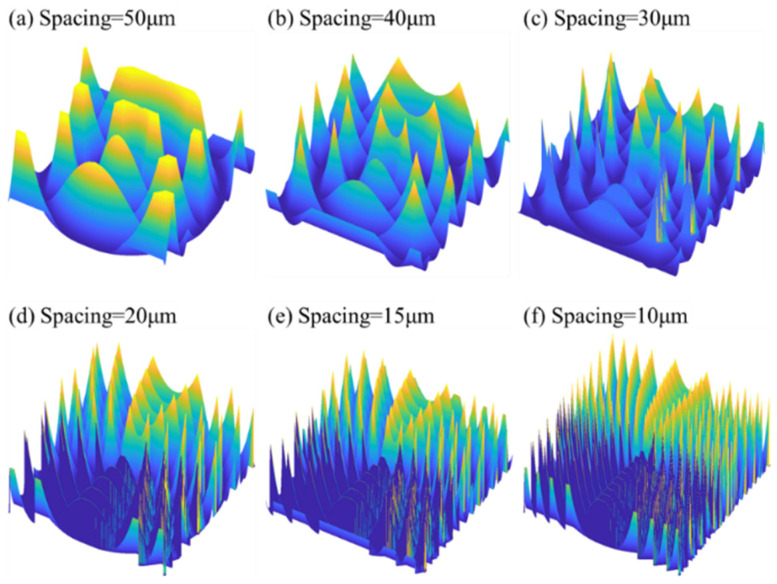
Comparison of simulated 3D surface topography of a polar microstructure with different groove spacing. Other important machining parameters: depth of cut: 5 μm: tool nose radius: 43 μm.

When decreasing GS, the simulation results show that there are more designed feature points in the FOV. However, the model does not consider the material properties of the workpiece. To clearly show the cusp caused by SPDT and SPDG, another series of simulations was conducted, whose spacings varied from 50 to 10 μm, as shown in Figure 19.

**Figure 20:**
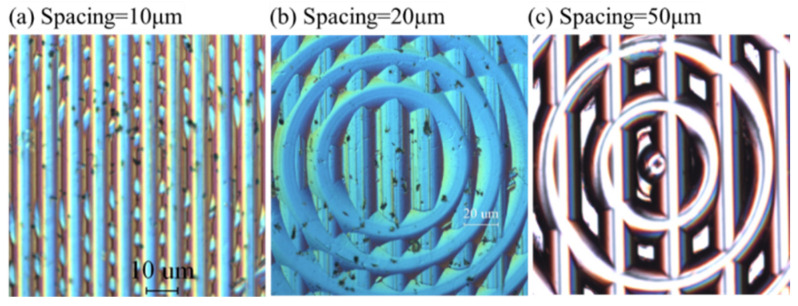
Experimental results of machining a polar microstructure with different groove spacing.

It was found that, when GS was 50 μm, the cusp was smooth, indicating that the material properties do not significantly influence the generation of polar microstructure. However, the cusp in the polar microstructure became large and more intensive when the GS decreased. From the view of the model simulation, they are cusps, but in real conditions, there are many more machining errors due to elastic deformation in ductile materials. In this study, three groups of polar microstructures were machined, as shown in Figure 20. The kinds of GS were 10 μm, 20 μm, and 50 μm. It is interesting to note from Figure 20a,b that the machining quality of polar microstructures was far from that of the simulation due to material properties, but when the GS increased to 50 μm, the machining quality was relatively consistent with the geometrical model. It can also be concluded that the range of GS should be around 50 μm after considering both function performance and machining quality.

On the whole, the machining parameters of polar microstructures were investigated in order to optimize their performance in feature point detection. The final machining parameters were chosen as referred to in this investigation and they are summarized in Table 1. This paper not only provides a turn-key solution for determining the optimal parameters but also presents a systematic way of studying the cutting strategy in ultra-precision machining of polar microstructures for optical precision measurement.

## 6. Conclusions

In this study, the influence of the cutting strategy on polar microstructures was investigated. Considering the FOV, the rough size range of grooving spacing was determined. The offset spacing was also designed for a 360-degree measurement. After that, the modeling results of the surface topography were compared with the measured result, which indicated that it is capable of using the simulated surface images for further processing, which greatly reduces the cost. A FRFP-based algorithm was introduced to detect the feature points, and the results show that the polar microstructure was well designed and the algorithm is suitable for detection. Lastly, a series of simulations and experiments was conducted to investigate the influence of machining parameters on the performance of polar microstructures. The three main parameters focused on were tool geometry, depth of cut, and groove spacing. Some experiments were also conducted to demonstrate the accuracy of the simulated results. The optimized parameters were finally chosen for further machining. Other machining parameters, such as cutting speed effect, should be further investigated in future work. This work contributes to the parameter optimization of optic-functional microstructure surface through both theoretical and experimental study.

## Figures and Tables

**Table 1:** Machining parameters for polar microstructures.

Machining straight grooves (SPDG)	Feed rate of grooving straight grooves (mm/min)	800
	DOC: um	5
	Δs (μm)	10
	$p_{S}$ (μm)	50
	Number of straight grooves:	250
Machining round grooves (SPDT)	DOC: um	5
	$p_{T}$ (μm)	50
	Number of round grooves:	250
	The radius of the smallest round groove	100
